# Hydrothermal synthesized delafossite CuGaO_2_ as an electrocatalyst for water oxidation

**DOI:** 10.1007/s12200-022-00014-7

**Published:** 2022-04-12

**Authors:** Han Gao, Miao Yang, Xing Liu, Xianglong Dai, Xiao-Qing Bao, Dehua Xiong

**Affiliations:** 1 State Key Laboratory of Silicate Materials for Architectures, Wuhan University of Technology, Wuhan, 430070 China; 2 Wuhan National Laboratory for Optoelectronics, Huazhong University of Science and Technology, Wuhan, 430074 China; 3 State Key Laboratory of Optical Technologies on Nanofabrication and Microengineering, Institute of Optics and Electronics, Chinese Academy of Sciences, Chengdu, 610209 China

**Keywords:** Hydrothermal, Water splitting, Delafossite, CuGaO_2_ (CGO), Electrocatalyst

## Abstract

Hydrogen production from water splitting provides an effective method to alleviate the ever-growing global energy crisis. In this work, delafossite CuGaO_2_ (CGO) crystal was synthesized through hydrothermal routes with Cu(NO_3_)_2_·3H_2_O and Ga(NO_3_)_3_·*x*H_2_O used as reactants. The addition of cetyltrimethylammonium bromide (CTAB) was found to play an important role in modifying the morphology of CuGaO_2_ (CGO-CTAB). With the addition of CTAB, the morphology of CGO-CTAB samples changed from irregular flake to typical hexagonal sheet microstructure, with an average size of 1–2 μm and a thickness of around 100 nm. Furthermore, the electrocatalytic activity of CGO-CTAB crystals for oxygen evolution reaction (OER) was also studied and compared with that of CGO crystals. CGO-CTAB samples exhibited better activity than CGO. An overpotential of 391.5 mV was shown to be able to generate a current density of 10 mA/cm^2^. The as-prepared samples also demonstrate good stability for water oxidation and relatively fast OER kinetics with a Tafel slope of 56.4 mV/dec. This work highlights the significant role of modification of CTAB surfactants in preparing CGO related crystals, and the introduction of CTAB was found to help to improve their electrocatalytic activity for OER.

**Graphical abstract:**

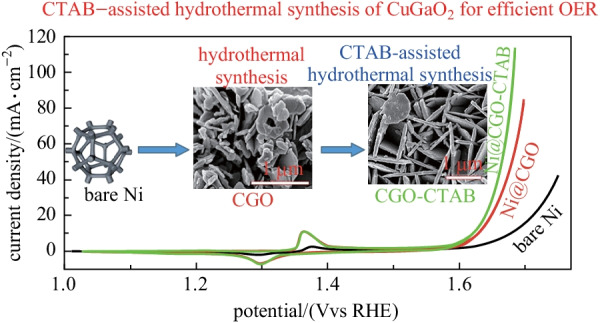

## Introduction

To alleviate the global energy crisis and environmental pollution, the development of clean energy storage and conversion devices is required [1–3]. Water electrolysis is one of the most important energy technologies for producing clean hydrogen fuel [4, 5]. The oxygen evolution reaction (OER) in water electrolysis is considered to be an important semi reaction for carbon dioxide electro-reduction, metal-air batteries, hydrogen production, and nitrogen electro-reduction, because the slow OER significantly affects the overall reaction efficiency of water electrolysis [6–8]. Currently, noble metal oxides [9–11] (such as RuO_2_ and IrO_2_) are still efficient catalysts for OER. Nevertheless, both the high cost of precious metals and the low catalytic efficiency of the oxides hinder their application. Hence it is necessary to develop efficient and earth abundant non-noble metal OER catalysts.

In recent years, many oxide-based electrocatalysts for water splitting, including transition metal oxides [12, 13], hydroxides [14, 15], selenides [16, 17], and phosphates [18, 19] have exhibited certain OER catalytic activities. A new class of transition metal oxides, delafossite materials (ABO_2_), have also shown great potential for electrocatalytic applications, such as CuFeO_2_ [20]. CuCoO_2_ [21–23], CuMnO_2_ [24]. Zhang et al. [25] have successfully customized the surface charge of silver delafossite AgCoO_2_ by adjusting the surface charge transfer state and transferring it into an efficient OER catalyst. Compared with pure AgCoO_2_ (395 mV), and RuO_2_ (369 mV) or IrO_2_ (338 mV) catalysts, AgCoO_2_/Ag catalysts need an overpotential of 271 mV to reach a current density of 10 mA/cm^2^. Mao et al. [24] synthesized CuMnO_2_ powder by sol–gel method. The CuMnO_2_ working electrode showed a current density of 12.3 mA/cm^2^ at 1600 r/min and 11.9 mA/cm^2^ without rotation for OER. In contrast to lots of work focusing on the application of p-type ABO_2_ oxides in water electrolysis, very few studies have paid attention to CuGaO_2_ (CGO) oxides. CGO is of great potential in catalytic materials because of its ultra-high carrier mobility, appropriate bandgap (~ 2.0 eV), and excellent long-term stability [26–28]. Ahmed and Mao [29] prepared three different morphologies of CGO, namely nanoparticles, submicron hexagons, and micron-sized particles, by a hydrothermal method at 190 °C, a sono-chemical method at 850 °C, and a solid-state reaction at 1150 °C, respectively. The CGO nanocrystals produce a current density of 23 mA/cm^2^ when the applied voltage is 0.6 V in 1 mol/L KOH solution for OER. They also successfully synthesized CGO nanoparticles at 850 °C by the sol–gel method [30]. The current densities of CGO nanoparticles were found to be 15 and 18 mA/cm^2^ for H_2_ and O_2_ generation, respectively, in 0.5 mol/L KOH solution.

Since 2015, our team has been working on the hydrothermal synthesis of oxides, and we have successfully synthesized CuAlO_2_ [31], CuCrO_2_ [32, 33], CuFeO_2_ [34, 35], CuMnO_2_ [36, 37], CuScO_2_ [38], CuCoO_2_ [21–23, 39, 40]. Du et al. [21] synthesized Ca doped CuCoO_2_ via a simple polyvinylpyrrolidone-assisted hydrothermal process, which required an overpotential of 470 mV to achieve a current density of 10 mA/cm^2^ and a small Tafel slope of 96.5 mV/dec in alkaline solution for OER. Deng et al. [38] synthesized CuScO_2_ by a hydrothermal method. The CuScO_2_ particle-loaded foam nickel electrode requires an overpotential of 490 mV to provide a current density of 10 mA/cm^2^ for OER. In this work, we intend to study the influence of the water oxidation performance of CGO with cetyltrimethylammonium bromide (CTAB) used as a surfactant in hydrothermal reaction. In detail, the effect of mineralizer on the synthesis of CGO was studied by changing the amount of NaOH at first, and then by adding a surfactant of CTAB to modify the morphology and structure of CGO crystals. Finally, CGO electrocatalyst with an excellent OER activity was obtained as expected.

## Experimental details

### Materials synthesis

All the chemicals in the experiment were of analytical grade and purchased from Sinopharm Chemical Reagent Co., Ltd without further treatment. CGO is synthesized by a one-step hydrothermal method, modified from our previous works [32, 33]. Usually, 10 mmol Cu(NO_3_)_2_·3H_2_O and 10 mmol Ga(NO_3_)_3_·*x*H_2_O were dissolved in 70 mL deionized solution and 5 mL ethylene glycol. Different amounts of NaOH (1.0, 2.0, 3.0 g NaOH), and surfactant (0, 1.8 g CTAB) were added to the above solution and stirred evenly. Then the solution was put into a 100 mL Teflon-lined autoclave and reacted in an oven at 190 °C for 24 h. The obtained precipitate was washed several times with ammonia, deionized water, and absolute ethanol, and then dried at 70 °C for 4 h for further characterization.

### Structural characterization

The crystal structure of the prepared CGO samples was analyzed by X-ray diffraction (XRD, D8 Advance, Bruker). The morphology, microstructure, and chemical composition of CGO powders were observed by using field-emission scanning electron microscopy (FESEM, S4800, Hitachi) coupled with energy-dispersive X-ray spectroscopy (EDS). The surface chemical states of CGO powders were analyzed by X-ray photoelectron spectroscopy (XPS, Thermo Escalab 250Xi), and the C 1s line (284.80 eV) corresponding to the surface adventitious carbon (C–C line bond) was used as the reference binding energy. The BET (Brunauer–Emmett–Teller) specific surface areas and porosity parameters of these CGO samples were measured by N_2_ adsorption–desorption isothermetry (Micromeritics TriStar II 3020 3.02).

### Electrochemical measurements

The three electrode system was used for the electrochemical performance test. The saturated calomel electrode and platinum wire were used as the reference electrode and counter electrode, respectively, and 1 mol/L KOH solution was used as the electrolyte. The working electrode was prepared as follows. Typically, 15 mg CGO powder was ultrasonically dispersed in 500 μL water, 480 μL isopropanol, and 20 μL Nafion solution to prepare CGO suspension. The 20 μL CGO suspension was dripped on nickel foam (1 cm^2^) with a pipette gun and then dried to obtain the working electrode, the loading mass of CGO electrocatalyst was 0.30 mg/cm^2^. The same loading mass of RuO_2_ electrode, supported by nickel foam, was fabricated as a reference sample. The electrocatalytic performance of OER was evaluated by cyclic voltammetry (*CV*), electrochemical impedance spectroscopy (EIS), and chronopotentiometry at room temperature (~ 25 °C) using a CS2350H electrochemical workstation (Wuhan Corrtest Instruments Corp., China). All current density values were normalized relative to the geometrical surface area of the working electrode. All *CV* curves presented in this work were IR-corrected. The electrochemical data processing methods were based on previous works [21–23].

## Results and discussion

**Fig. 1:**
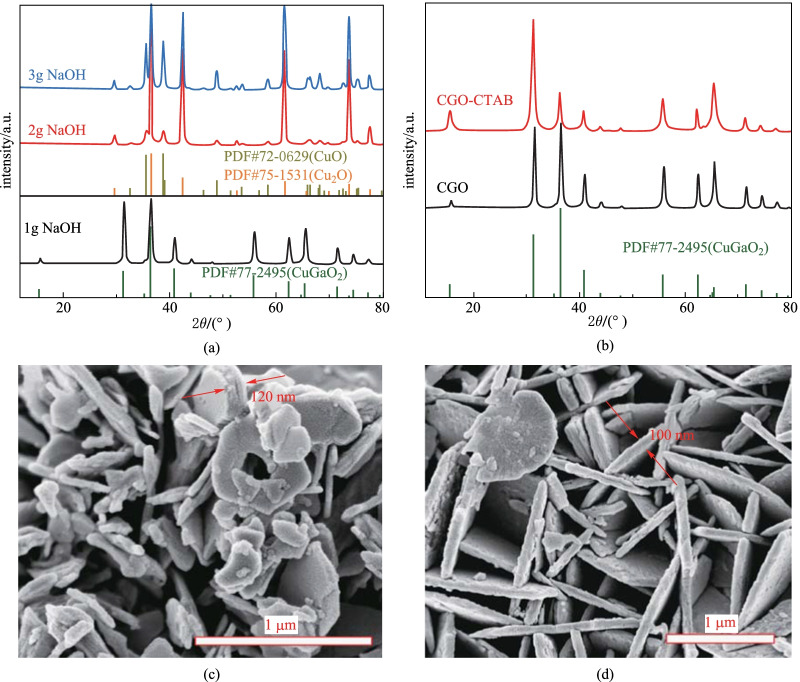
**a** and **b** XRD patterns and SEM images (**c** CGO, **d** CGO-CTAB) of CGO samples

In our previous reports [21, 37, 38, 41], the concentration of NaOH in the precursor solution was the key factor affecting the crystal growth of ABO_2_ oxides during hydrothermal synthesis. Using Cu(NO_3_)_2_·3H_2_O and Ga(NO_3_)_3_·*x*H_2_O as reactants, the amount of mineralizer NaOH (samples No. 1, No. 2, No. 3 in Table 1) was adjusted to prepare CGO crystals. As shown in Fig. 1a, when the amount of NaOH was 2 and 3 g, the majority of the XRD diffraction peaks of the as-obtained product matched well with CuO (JCPDS card No. 72-0629) and Cu_2_O (JCPDS card No. 77-1531) phases, and the other weak diffraction peaks could be indexed to CGO (JCPDS card No. 77-2495). Fortunately, the pure phase of CGO crystal was achieved when the addition amount of NaOH was 1 g (denoted as CGO). In detail, the strong diffraction peaks at 2*θ* values of 15.5°, 31.2°, 36.4°, 40.8°, 55.7°, 62.3°, and 65.2° correspond to the (003), (006), (012), (104), (018), (110), and (1010) planes of the CGO, respectively, without any other impurities. The corresponding SEM image (Fig. 1c) shows that the size of CGO crystals is not uniform, ranging from 200 to 600 nm. The thickness of CGO particles is also uneven, up to 120 nm. It is known that the activity of the electrocatalyst is greatly influenced by its morphology and active sites [42]. The smaller the CGO particles are, the larger the specific surface area is, and the more active sites are exposed. Generally, the surfactant used in the hydrothermal reaction can help to control the particle size and the morphology of the product. By binding on the particle surface, surfactant molecules create a barrier for crystals proximity and induce strong spatial repulsion [43, 44]. To optimize the structure and morphology of CGO, 5 mmol CTAB was added to improve the reaction kinetics and reduce the agglomeration of CGO particles (No. 4 in Table 1). XRD analysis in Fig. 1b confirms the formation of the CGO phase when 5 mmol CTAB (denoted as CGO-CTAB) was added in hydrothermal precursors. The CGO-CTAB samples (Fig. 1d) exhibit a typical hexagonal morphology with a thickness of about 100 nm and a transverse size around 1–2 μm. After adding the CTAB surfactant, the morphology and size of CGO particles become more uniform.

**Table 1:** Details of the reaction conditions employed to synthesize CGO crystals

Sample	Reactant	Solvent	EG/mL	CTAB/mmol	NaOH/g	Temperature/°C and time/h
No. 1	10 mmol Cu (NO_3_)_2_ + 10 mmol Ga (NO_3_)_3_	70 mL Deionized water	5	0	1.0	
No. 2					2.0	190 °C
No. 3					3.0	24 h
No. 4				5	1.0

**Fig. 2:**
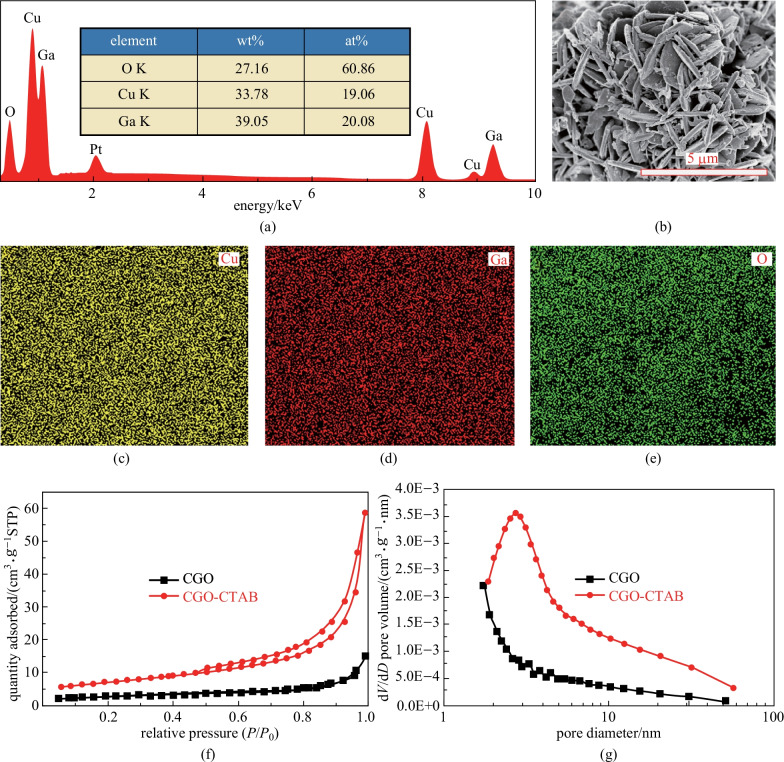
**a** Elemental analysis report, **b** SEM image, **c**–**e** EDS elemental mappings, **f** N_2_ adsorption–desorption, and **g** corresponding pore size distribution plots of CGO samples

In addition, the chemical composition of CGO-CTAB samples was also studied. The SEM–EDS spectrum in Fig. 2a shows that there are four elements (Cu, Ga, O, Pt) in CGO-CTAB powders, of which Pt comes from platinum powder sprayed to enhance the conductivity of the sample before the SEM test. The atomic ratio of Cu/Ga is 19.06:20.08 (1:1.05) for the CGO-CTAB samples, which is very consistent with the stoichiometric ratio. Figure 2c–e suggests a uniform distribution of the Cu, Ga, and O elements in the CGO-CTAB nanoplates. The nitrogen adsorption–desorption isotherms of as-prepared CGO powders are presented in Fig. 2f–g. The absorption isotherms of CGO and CGO-CTAB samples showed typical type IV adsorption isotherms and hysteresis rings, indicating the existence of typical mesoporous structures [13, 45, 46]. CGO-CTAB samples showed a high specific surface area, 24.69 m^2^/g, which is larger than that of CGO (9.50 m^2^/g) in this work and CuFeO_2_ (11.38 m^2^/g) in Ref. [47]. The high specific surface area of CGO-CTAB provides a large electrode–electrolyte interface for the reactants and mesoporous morphology can provide developed transportation channels for water electrocatalytic reactants [46]. Besides, the pore volume of CGO-CTAB (0.091 cm^3^/g) is larger than that of CGO (0.023 cm^3^/g). The high porosity of catalysts is conducive to the separation of reactants and products during the OER process [48]. The pore size distribution of CGO-CTAB is centered around 2 nm in the mesoporous range according to the BJH models (Fig. 2 g), which is in favor of the escape of newly generated oxygen gas molecules. These results show that CGO-CTAB with a large number of mesopores results in a large specific surface area and can provide more catalytic active sites, thereby helping to improve their electrocatalytic performance.

**Fig. 3:**
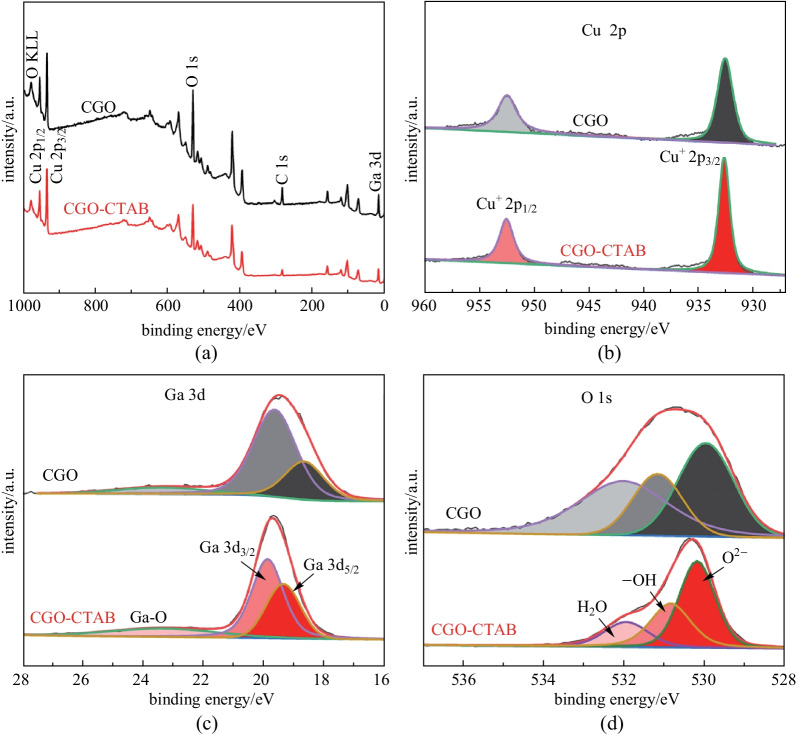
XPS spectra of CGO and CGO-CTAB sample: **a** survey scan, **b**–**d** high-resolution scans for Cu 2p, Ga 3d, and O 1s

The chemical structures and surface chemical states of the CGO and CGO-CTAB samples were further investigated via XPS. Figure 3a exhibits typical signals of C 1s, O 1s, Cu 2p, and Ga 3d based on the XPS survey spectrum, indicating the presence of Cu, Ga, and O in CGO and CGO-CTAB samples. As shown in Fig. 3b–d, the high resolution spectra of Cu 2p, Ga 3d, and O 1s signals of CGO and CGO-CTAB samples were obtained to study their chemical states. Figure 3b displays the two strong binding energy (BE) peaks at around 932.6 and 952.4 eV which can be attributed to Cu 2p_3/2_ and Cu 2p_1/2_, respectively, suggesting the existence of monovalent copper cations (Cu^+^) in CGO and CGO-CTAB [49–51]. This is consistent with our previous reports on other delafossite oxides CuCoO_2_ [21], CuScO_2_ [41], and CuMnO_2_ [37]. Figure 3c presents a high resolution Ga 3d spectrum for the CGO and CGO-CTAB, which consists of two main broad peaks corresponding to Ga 3d_5/2_ and Ga 3d_3/2_ spin–orbit lines. The CGO and CGO-CTAB samples have two prominent peaks at BE of 18.7 and 19.6 eV which can be ascribed to the Ga 3d_5/2_ and Ga 3d_3/2_, respectively, indicating the presence of Ga^3+^ chemical state [52, 53]. While the other peaks located at 23.3 eV of BE correspond to the Ga-O bond [54, 55]. The high resolution spectra of the O 1s spectrum (Fig. 3d) can be resolved into three peaks. The peaks at 530.0 eV correspond to the O^2−^ (lattice oxygen) of the delafossite oxides (CGO and CGO-CTAB) [56, 57]. The BE at around 531.2 eV supports the -OH (oxygen in a hydroxyl group) [58, 59], which is considered as the highest active oxygen, able to improve the generation of active species in the OER process [50]. The fitting peaks at 532.0 eV were assigned to the H_2_O (oxygen of physically absorbed H_2_O molecules) on CGO and CGO-CTAB samples [60, 61].

**Fig. 4:**
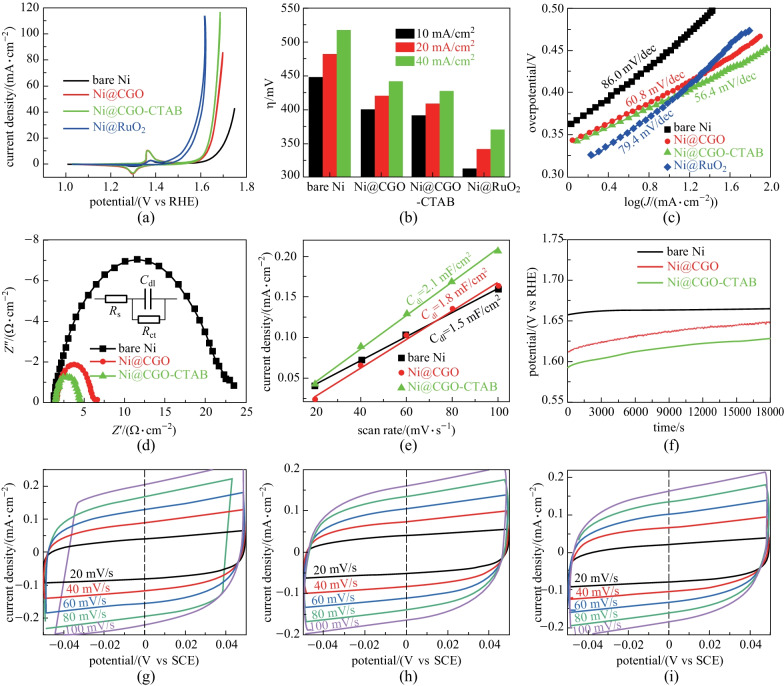
**a** *CV* curves; **b** overpotentials needed to deliver anodic current density of 10, 20, and 40 mA/cm^2^; and **c** Tafel plots of bare Ni, Ni@RuO_2_, Ni@CGO, and Ni@CGO-CTAB working electrodes; **d** Nyquist plots; **e** double-layer capacitance *C*_dl_ and **f** chronopotentiometric curves of bare Ni, Ni@CGO, and Ni@CGO-CTAB working electrodes; *CV* curves of **g** bare Ni, **h** Ni@CGO, and **i** Ni@CGO-CTAB working electrodes measured in 1.0 mol/L KOH in the non-Faradaic region with different scan rates from 20 to 100 mV/s. The inset in panel (**d**) shows the equivalent circuit model for these CGO based electrodes

The electrocatalytic activity of CGO for OER was evaluated by *CV* in 1.0 mol/L KOH. Figure 4a shows the *CV* curves of the two electrodes loaded with different CGO powder and these two electrodes are expressed as Ni@CGO, Ni@CGO-CTAB, respectively. For comparison, the OER performances of the bare nickel foam (denoted as bare Ni) and the nickel foam loaded with commercial RuO_2_ powder (denoted as Ni@RuO_2_) were also tested. The corresponding overpotentials of these four electrodes at different current densities were obtained from the reduction branches of the *CV* curves as shown in Fig. 4b. As seen in Table 2, the OER polarization curves of bare Ni achieved a current density of 10 mA/cm^2^ at a low overpotential of 448.6 mV. After loading with CGO powder, Ni@CGO and Ni@CGO-CTAB exhibited overpotentials of 399.6 and 391.5 mV, respectively, while Ni@RuO_2_ only required a low overpotential of 312.5 mV. Compared with the bare Ni (*η*_20_ = 481.9 mV) and Ni@CGO (*η*_20_ = 419.9 mV), Ni@CGO-CTAB (*η*_20_ = 408.9 mV) showed lower overpotentials at 20 mA/cm^2^. To understand the underlying dynamics of OER, Tafel analysis curves (Fig. 4c) were obtained by processing partial reduction curves in *CV*. The Tafel slopes of Ni@CGO and Ni@CGO-CTAB are 60.8 and 56.4 mV/dec, respectively, which are much smaller than those of the bare Ni (86.0 mV/dec) and Ni@RuO_2_ (79.4 mV/dec). The smaller the Tafel slope, the easier the oxygen evolution. Thus, the reaction kinetics of Ni@CGO-CTAB is the best and the electrocatalytic speed is the fastest. Moreover, the overpotential and Tafel slope of the CGO-CTAB electrode is lower than those of some other non-noble metal oxide catalysts (shown in Table 2), for example, delafossite oxide CuCoO_2_ (*η*_10_ = 440 mV, Tafel slope = 92.8 mV/dec) [22], AgCoO_2_ (*η*_10_ = 395 mV) [25], CGO nanocrystals (*η*_23_ = 0.6 V vs Ag/AgCl) [29], CGO micro-sized particles nanocrystals (*η*_5_ = 0.6 V vs Ag/AgCl) [29], CuCoO_2_ (*η*_10_ = 390 mV, Tafel slope = 70 mV/dec) [23], Ca doped CuCoO_2_ (*η*_10_ = 470 mV, Tafel slope = 96.5 mV/dec) [21], and CuScO_2_ (*η*_10_ = 470 mV, Tafel slope = 114 mV/dec) [38], or other perovskite electrocatalysts LaNiO_3_ (*η*_10_ = 550 mV, Tafel slope = 148 mV/dec) [62], LaNi_0.85_Mg_0.15_O_3_ (*η*_10_ = 450 mV, Tafel slope = 95 mV/dec) [62], LaFeO_3_ (*η*_10_ = 420 mV, Tafel slope = 62 mV/dec) [63].

**Table 2:** OER activity of these CGO based electrodes in this work in comparison to that of other delafossite oxides as well as other perovskite oxides OER catalysts recently reported in the literature

Electrodes	*η*_10_/mV	*η*_20_/mV	*η*_40_/mV	Tafel slope/(mV⋅dec^−1^)	*C*_dl_/(mF⋅cm^−2^)	*R*_ct_/Ω	Refs.
Bare Ni	448.6	481.9	517.1	86.0	1.5	21.2	This work
Ni@CGO	399.6	419.9	442	60.8	1.8	4.7	
Ni@CGO-CTAB	391.5	408.9	427.1	56.4	2.1	3.1	
GC@CuCoO_2_	440	–	–	92.8	2.5	65.4	[22]
GC@AgCoO_2_	395	–	–	–	–	–	[25]
Ni@CuScO_2_	470	510	–	114	–	–	[38]
GC@CuCoO_2_	390	410	–	70	6.6	2.9	[23]
Ni@CuCoO_2_	470	–	–	96.5	7.0	1.09	[21]
GC@LaNiO_3_	550	–	–	148	–	–	[62]
GC@LaNi_0.85_Mg_0.15_O_3_	450	–	–	95	–	–	[62]
GC@LaFeO_3_	420	–	–	62	–	–	[63]

During the OER process, the transport rate of ions and charge is one of the important factors affecting the kinetics of electrocatalysis. The EIS measurements were performed in the frequency range of 20 mHz–200 kHz under a constant potential of 1.60 V vs RHE. In the Nyquist diagram (Fig. 4d), the intersection of the high-frequency range curve and the real axis is the electrolyte resistance (*R*_s_), and the diameter of the semicircle is the charge transfer resistance (*R*_ct_); the equivalent circuit is shown in the inset of Fig. 4d. The *R*_s_ values of the bare Ni (*R*_s_ = 1.3 Ω), Ni@CGO (*R*_s_ = 1.5 Ω), and Ni@CGO-CTAB (*R*_s_ = 1.3 Ω) were similar. Furthermore, the bare Ni electrode had the largest charge transfer resistance (*R*_ct_ = 21.2 Ω), and the Ni@CGO-CTAB (*R*_ct_ = 3.1 Ω) and Ni@CGO (*R*_ct_ = 4.7 Ω) are smaller. Therefore, the Ni@CGO-CTAB showed the fastest charge transfer rate in the OER rate control step, which is conducive to the rapid charge transfer between the KOH solution and electrode interface. This result is consistent with the above analysis result of overpotential and Tafel slope.

The electrochemical active surface area (ECSA) is another important factor in the analysis of electrocatalytic activity. ECSA is proportional to the electrochemical double-layer capacitance (*C*_dl_) of the catalyst surface [1, 46, 64]. As shown in Fig. 4 g–i, at the same scanning speed the *CV* curve area and current density of Ni@CGO-CTAB are larger. The calculated *C*_dl_ value (Fig. 4e) of Ni@CGO-CTAB (2.1 mF/cm^2^) is higher than that of the original value Ni@CGO (1.8 mF/cm^2^) and bare Ni (1.5 mF/cm^2^). The result is in good agreement with BET results, which indicates that CGO-CTAB has a larger effective electrochemical area with more OER active sites.

**Fig. 5:**
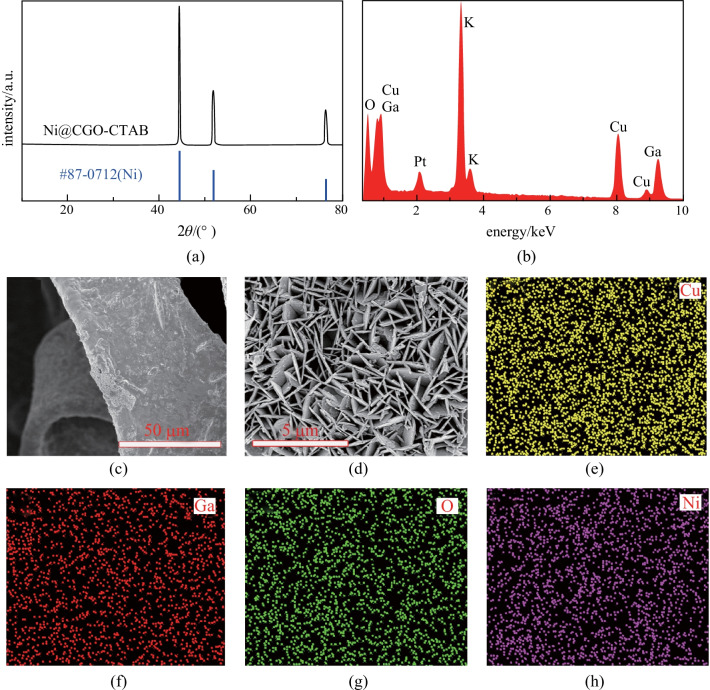
**a** XRD pattern; **b** EDS spectrum result; **c** and **d** SEM images; and elemental mappings (**e** Cu; **f** Ga; **g** O; **h** Ni) of Ni@CGO-CTAB working electrode after long-term stability test for 5 h under OER condition

Stability is another important index to evaluate the catalytic performance of electrocatalyst since the stability of the material can reflect its practical application value. As shown in Fig. 4f, the stability of the material was tested for 18,000 s at a constant current density of 5 mA/cm^2^. The initial potentials of Ni@CGO and Ni@CGO-CTAB electrodes were 1.61 and 1.59 V vs RHE, respectively, consistent with the *CV* results (Fig. 4a). During the whole electrolysis process, the potential of the bare Ni electrode did not increase obviously (1.66 V vs RHE). However, the required potential of both Ni@CGO and Ni@CGO-CTAB electrodes increased about 35 mV. These two electrodes loaded with CGO powder showed slight degradation, which may be related to the detachment of CGO powder from foam nickel. The XRD pattern in Fig. 5a of Ni@CGO-CTAB working electrode after the stability test can be indexed to the pure phase Ni (JCPDS card No. 87–0712). Due to the low loading mass, there is no diffraction signal belonging to the CGO crystal. Figure 5b exhibits the chemical composition of Ni@CGO-CTAB after the stability test, confirming the coexistence of Cu, Ga, O, K, and Ni, in which K comes from OER electrolyte and Ni comes from nickel foam. After extended OER electrolysis for durability test, the CGO-CTAB sample (Fig. 5c, d) retains a typical hexagonal nanosheet morphology. EDS mapping results in Fig. 5e–h indicates that the Cu, Ga, O, and Ni elements are distributed uniformly in the sample. It is noteworthy that the above results indicate that the changes of morphology and chemical composition of CGO-CTAB in the water oxidation process can be ignored, even after an extended OER electrolysis for 18,000 s.

## Conclusions

In summary, CuGaO_2_ (CGO) crystals with a uniform size were obtained by a hydrothermal reaction at 190 °C for 24 h, and were prepared with Cu(NO_3_)_2_, Ga(NO_3_)_3_, NaOH, and cetyltrimethylammonium bromide as reactants. The introduction of CTAB not only modified the morphology and surface states through the interaction between surfactant and CGO microstructure but also affected the electrocatalytic activity of OER. Compared with CGO samples without surfactant, CGO-CTAB samples demonstrated a more uniform size (transverse size 1–2 μm, thickness ~ 100 nm), larger specific surface area (24.69 m^2^/g), and higher OER catalytic activity (a lower overpotential of 391.5 mV at 10 mA/cm^2^ and a smaller Tafel slope of 56.4 mV/dec). After a long-term stability test for 18,000 s under OER condition, the Ni@CGO-CTAB electrode still maintained excellent OER catalytic activity, and the potential degradation was only 36 mV. Therefore, the as-prepared delafossite CGO-CTAB has good electrocatalytic activity and durability, which show its broad application prospect for water oxidation.
